# Targeting Glucose Metabolism to Enhance Immunotherapy: Emerging Evidence on Intermittent Fasting and Calorie Restriction Mimetics

**DOI:** 10.3389/fimmu.2019.01402

**Published:** 2019-06-25

**Authors:** William J. Turbitt, Wendy Demark-Wahnefried, Courtney M. Peterson, Lyse A. Norian

**Affiliations:** ^1^Nutrition Obesity Research Center, University of Alabama at Birmingham, Birmingham, AL, United States; ^2^Department of Nutrition Sciences, University of Alabama at Birmingham, Birmingham, AL, United States; ^3^O'Neal Comprehensive Cancer Center, University of Alabama at Birmingham, Birmingham, AL, United States

**Keywords:** immunotherapy, immune checkpoint blockade, tumor immunology, caloric restriction, calorie restriction mimetics, intermittent fasting, fasting-mimicking diet, time-restricted feeding

## Abstract

There is growing interest in harnessing lifestyle and pharmaceutical interventions to boost immune function, reduce tumor growth, and improve cancer treatment efficacy while reducing treatment toxicity. Interventions targeting glucose metabolism are particularly promising, as they have the potential to directly inhibit tumor cell proliferation. However, because anti-tumor immune effector cells also rely on glycolysis to sustain their clonal expansion and function, it remains unclear whether glucose-modulating therapies will support or hinder anti-tumor immunity. In this perspective, we summarize a growing body of literature that evaluates the effects of intermittent fasting, calorie restriction mimetics, and anti-hyperglycemic agents on anti-tumor immunity and immunotherapy outcomes. Based on the limited data currently available, we contend that additional pre-clinical studies and clinical trials are warranted to address the effects of co-administration of anti-hyperglycemic agents or glucose-lowering lifestyle modifications on anti-tumor immunity and cancer treatment outcomes. We stress that there is currently insufficient evidence to provide recommendations regarding these interventions to cancer patients undergoing immunotherapy. However, if found to be safe and effective in clinical trials, interventions targeting glucose metabolism could act as low-cost combinatorial adjuvants for cancer patients receiving immune checkpoint blockade or other immunotherapies.

## Introduction

Cancer encompasses a broad family of diseases that involve abnormal and unregulated cell proliferation. Hanahan and Weinberg (1) have detailed the underlying characteristics that all cancers possess, including sustained proliferative signals, dysregulated cellular energetics, avoidance of immune-mediated killing, tumor-promoting inflammation, invasion, and metastasis. These factors promote a feed-forward loop favoring an immune-evading microenvironment that supports tumor progression. The balance between protective anti-tumor mechanisms and tumor-promoting/immunosuppressive factors is critical for dictating cancer progression or remission (2).

### Can Glucose Metabolism be Targeted to Improve Immunotherapies?

Cancer immunotherapies are designed to enhance the protective immune responses that can eliminate established tumors and are promising treatment options for many cancers. Cancer immunotherapy comprises multiple strategies, including cytokine therapies, targeted antibodies, adoptive cell transfers, genetically engineered chimeric antigen receptor (CAR) T cells, cancer vaccines, genetically engineered oncolytic viruses, and immune checkpoint blockade (ICB). Successes have been observed within each category; however, ICB-based therapies are the most frequently utilized immunotherapy and are currently FDA-approved as treatment options in patients with many types of advanced cancers. ICB uses antibodies to disrupt the receptor/ligand pairs that send inhibitory signals to effector T cells (e.g., Programmed Death-1 [PD-1] and Programmed Death-Ligand 1 [PD-L1]) (3). Despite demonstrated clinical benefit, typically <50% of patients receiving ICB experience objective, durable responses (4, 5). This challenge has led to a major push to improve ICB efficacy by developing novel combinatorial treatment strategies to reduce cancer cell viability and proliferation, increase tumor infiltration by effector T cells, and/or promote T cell effector function in the tumor microenvironment.

One combinatorial approach that has garnered much attention in recent years is the use of glucose-limiting lifestyle changes or anti-diabetic drugs (summarized in Table 1) that can be co-administered with immunotherapy. The rationale underlying this approach is that tumor cells are often dependent on glucose as a primary energy source. This glycolytic dependency arises from the continuous proliferation of tumor cells, which necessitates uninterrupted access to energy and the building blocks of cellular biomass. To meet these requirements, cancer cells utilize glycolysis, even in the presence of oxygen, a process referred to as aerobic glycolysis or the “Warburg effect” (6). Thus, lifestyle and pharmacologic interventions that reduce intra-tumoral glucose levels may slow cancer cell replication and render cancer cells more susceptible to immune-mediated killing, thereby boosting the effectiveness of immunotherapy.

**Table 1:** Defining lifestyle interventions and pharmaceutical agents.

**Intervention**		**Definition**
Continuous calorie restriction		A chronic state in which caloric intake is less than caloric need by reducing daily energy intake by a minimum of 10-20%.
Intermittent fasting approaches	Intermittent energy restriction	Restricting energy intake to ~60–75% below energy requirements for short periods, followed by periods with normal energy intake (e.g., the 5:2 diet [consisting of ~5 days of eucaloric feeding and ~2 days of a very-low-calorie diet per week]).
	Short-term fasting	Temporarily fasting, typically for a period between 24 and 48 h.
	Fasting-mimicking diet	Maintaining a fasting-like state by periodically consuming a very-low-calorie, low-protein diet (not necessarily fasting).
	Time-restricted feeding	Reducing food intake to a set number of hours each day (e.g., eating in a <10 h daily period).
Ketogenic diet		An ultra-low carbohydrate diet (typically ~5% of kcal) that does not directly restrict calories or require periods of fasting. Successful generation of ketone bodies can suppress appetite and reduce plasma glucose concentrations in cancer-free individuals.
Caloric restriction mimetics (e.g., Hydroxycitrate, Resveratrol)		Compounds that mimic the beneficial effects of caloric restriction.
Anti-hyperglycemic agents (e.g., Metformin)		Agents that lower glucose levels in the blood and are often used to treat type 2 diabetes mellitus.

A major concern with any treatment approach focused on limiting glucose availability is that it may have unintended negative consequences for protective immunity. This is because effector CD8^+^ T cells also rely on glucose-dependent, Warburg-style metabolism for their clonal expansion and anti-cancer functions, including cytolytic activity and cytokine secretion (7). Prior studies report that dysregulated CD8^+^ T cell metabolism within the tumor microenvironment impairs T cell effector functions and promotes tumor progression (8, 9). For instance, in treatment-naive human subjects with clear cell renal cell carcinoma, tumor-infiltrating CD8^+^ T cells exhibit a loss of proliferative capacity due to metabolic defects, including impaired glucose uptake and glycolytic capacity; fragmented and hyperpolarized mitochondria; and increased production of reactive oxygen species (10). These observations lend validity to concerns that further limitations of intra-tumoral glucose will impair both T cell and tumor cell metabolism. However, an elegant study by Chang et al. provided evidence that ICB may selectively protect T cells from reduced glucose availability within the tumor microenvironment (8). In this report, the authors illustrated that ICB administration with either anti-CTLA-4, anti-PD-1, or anti-PD-L1 *improved* the glycolytic capacity and Interferon-gamma (IFNγ) production of CD8^+^ tumor-infiltrating T cells (8). The same study determined that anti-PD-L1 *inhibited* glucose uptake and glycolysis in tumor cells. Therefore, ICB may differentially alter the metabolic programming of tumor cells vs. anti-tumor immune cells to favor cancer regression. This observation makes ICB a particularly attractive type of immunotherapy to combine with glucose-limiting lifestyle interventions or anti-diabetic drugs, as the result may be impaired tumor cell metabolism and viability, with concomitantly improved T cell metabolism and effector function.

However, it remains unclear whether interventions that lower plasma glucose exert a net positive or negative effect on tumor proliferation, anti-tumor immunity, and cancer immunotherapy outcomes, particularly in the context of ICB. Minimal pre-clinical data exists, and no clinical trials have been conducted to determine if glucose-limiting lifestyle interventions or anti-diabetic drugs interact with other immunotherapy platforms, like adoptive cell therapies, cancer vaccines, or CAR T cells. These immunotherapy strategies may drive an immunometabolic profile more susceptible to reductions in glucose availability; therefore, broad-sweeping conclusions cannot be drawn on the applicability and safety of glucose-targeting therapies as an adjuvant to all immunotherapy strategies. Below, we review pre-clinical data regarding the effects of glucose-lowering interventions on tumor cell proliferation and anti-tumor immunity. Several reports have indicated that glucose-regulatory interventions may actually improve the efficacy of ICB and possibly other types of immunotherapy. When available, we also provide information about human subject data or ongoing clinical trials that are investigating these interventions in cancer patients. In light of the growing use of anti-hyperglycemic agents and surging popular interest in intermittent fasting and calorie restriction mimetics, we focus our discussion on this subset of promising interventions. Although other targeted therapies, like tyrosine kinase inhibitors (e.g., PI3K inhibitors), are promising for modulating signaling cascades relevant to glucose metabolism and for impacting immune responses following immunotherapy (11), these interventions were not discussed here because their primary modes of action are not glucose regulation.

## Calorie Restriction (CR)

CR is typically defined as a reduction in daily energy intake of at least 10–20% below regular *ad libitum* feeding, without inducing malnutrition (Table 1). CR has been explored in pre-clinical and clinical studies for its ability to extend lifespan and improve cardiometabolic health and is now being explored for its anti-cancer properties.

### Pre-clinical Findings

Abundant evidence from animal models demonstrates that CR reduces cancer incidence and delays cancer progression through multiple mechanisms (12–14). For example, CR can impair cancer cell proliferation by reducing plasma glucose and insulin, which in turn alters expression of cell cycle proteins, modifies tumor suppressor gene function, and disrupts metabolic pathways (15). CR can also reduce insulin-like growth factor-1 (IGF-1), a nutrient-sensing growth factor that is stimulated by glucose (16, 17). IGF-1 activates phosphoinositide 3-kinase (PI3K)/protein kinase B (Akt)/ mammalian target of rapamycin 1 (mTORC1) signaling pathways in cancerous cells to promote glycolysis and tumor cell proliferation, while simultaneously inhibiting apoptosis (17–20). Thus, the pleiotropic effects of CR converge to blunt the proliferative capacity of tumor cells. Pre-clinical data suggest that CR can sensitize cancerous cells to radiotherapy and chemotherapy by negatively regulating anti-apoptotic defense mechanisms (15, 21, 22). Additionally, Farazi et al. reported that chronic CR preserved antigen-specific CD4^+^ T cell priming and induced a significant survival benefit when combined with anti-OX40 (CD134) immunotherapy in aged tumor-bearing mice (23). Therefore, CR appears to both inhibit tumor cell proliferation and maintain anti-tumor immunity and has the potential to be combined with immunotherapy based on this pre-clinical finding.

#### Clinical Findings

Despite the potential to enhance immunotherapies, concerns about loss of lean mass and aversion to CR limit therapeutic translation to cancer patients who may already be struggling with cachexia and loss of appetite. Beneficial effects have been observed in an adjuvant setting when combined with targeted therapy or chemotherapy (15); however, to date, there have been no trials examining the effects of CR on ICB in humans (Table 2). Therefore, it is not clear whether CR can be safely combined with ICB or other immunotherapies to improve patient outcomes. Given the possibility for CR to accelerate cachexia in cancer patients, such studies should be approached with caution.

**Table 2:** Ongoing or completed clinical trials investigating lifestyle or pharmaceutical agents targeting glucose metabolism in combination with immunotherapy.

**Intervention**		**Drug treatment(s)**	**Disease(s)**	**NCT reference**	**Outcome/anticipated completion date**
Continuous calorie restriction		N/A	N/A	N/A	N/A
Intermittent fasting approaches	Intermittent energy restriction	Chemotherapy	Breast and ovarian cancer	NCT03162289	Recruiting, May 2020
	Short-term fasting	N/A	N/A	N/A	N/A
	Fasting-mimicking diet	Chemo-immunotherapy (carboplatin/ pemetrexed and pembrolizumab)	Non-small cell lung cancer	NCT03700437	Not yet recruiting
	Time-restricted feeding	N/A	N/A	N/A	N/A
Ketogenic diet		N/A	N/A	N/A	N/A
Caloric restriction mimetics (e.g., Hydroxycitrate, Resveratrol)		N/A	N/A	N/A	N/A
Anti-hyperglycemic agents (e.g., Metformin)		Nivolumab+Metformin	Non-small cell lung cancer	NCT03048500	Recruiting, Feb 2021

*The following online clinical trial registries were searched:*
World Health Organization—International Clinical Trials Registry Platform (http://apps.who.int/trialsearch/), U.S. National Library of Medicine (https://clinicaltrials.gov/), Health Canada Clinical Trial Database (https://health-products.canada.ca/ctdb-bdec/index-eng.jsp), European Union Clinical Trials Register (https://www.clinicaltrialsregister.eu/ctr-search/search), Australian New Zealand Clinical Trials Registry (http://www.anzctr.org.au/BasicSearch.aspx), Chinese Clinical Trial Registry (http://www.chictr.org.cn/searchprojen.aspx), Japan Primary Registries Network (https://upload.umin.ac.jp/cgi-open-bin/ctr_e/index.cgi?function=02).

## Intermittent Fasting

Alternatives to CR include intermittent fasting approaches such as short-term fasting, the fasting-mimicking diet (e.g., periodically consuming a very-low-calorie, low-protein diet to mimic a fasting-like state), and time-restricted feeding (eating in a ≤ 10-h daily period) (Table 1).

### Pre-clinical Findings

Intermittent fasting interventions (including intermittent energy restriction) have been shown repeatedly to reduce glycemia, improve insulin sensitivity, and reduce whole-body cell proliferation rates in studies of both tumor-free animals and humans (24–29)—suggesting that these approaches target glycemic pathways and have anti-proliferative effects. Studies in tumor-bearing mice show that fasting for 72 h reduces plasma glucose concentrations by ~40% and IGF-1 by ~70% (30). The fasting-mimicking diet can achieve similar reductions in plasma glucose and IGF-1 without the need for a prolonged fast (26). Thus, intermittent fasting may provide several of the same mechanistic benefits as CR, while mitigating adverse outcomes on lean mass.

A recent literature review of pre-clinical models by Lv et al. (13) reports that intermittent fasting approaches were significantly preventive against cancer in ~60% of animal studies. Both the fasting-mimicking diet and time-restricted feeding have been associated with improved cancer treatment outcomes in *all* animal models of cancer tested thus far, through immunologic and metabolic mechanisms similar to those induced by CR (31–34). In particular, these two interventions have improved responses to anthracycline-based chemotherapies, leading to decreased tumor outgrowth in murine models of sarcoma, lung, colon, melanoma, and breast cancer (31, 32). Delayed tumor growth was dependent upon increased percentages of intra-tumoral cytotoxic CD8^+^ T cells, with concomitant reductions in regulatory T cells, and elevated expression of the stress-responsive protein heme-oxygenase-1 (HO-1) in the tumor microenvironment. Importantly, key findings, such as increased tumor immunogenicity in mice on the fasting-mimicking diet, could be achieved simply by culturing tumor cells in low glucose culture medium prior to tumor challenge, which mirrors the decreased glucose concentrations achieved *in vivo* with this dietary intervention (30, 32). These data suggest that limited glucose availability stresses tumor cells and ultimately may promote stronger anti-tumor immunity. Of note, anthracyclines such as mitoxantrone and doxorubicin are known to cause immunogenic tumor cell death (35), explaining their ability to act in concert with fasting-based dietary changes to improve anti-tumor immunity. At this time, no pre-clinical studies have examined the ability of intermittent fasting to enhance the efficacy of ICB or other immunotherapies.

### Clinical Findings

Currently, there remains a shortage of clinical data investigating the effects of intermittent fasting on ICB efficacy in humans. To date, only one clinical trial (ISRCTN77916487) (Table 2) has investigated the effects of intermittent fasting (the 5:2 diet consisting of ~5 days of eucaloric feeding and ~2 days of a very-low-calorie diet per week) on cancer-related pathways in individuals at high risk for breast cancer (36). Although the cohort size was limited (*n* = 24), 55% of women showed evidence of reduced glycolysis, gluconeogenesis, glycogen synthesis, and lipid synthesis, suggesting that intermittent energy restriction altered glucose metabolism and decreased anabolic gene expression in over half of patients. Changes in breast epithelial cell differentiation were observed in some patients, but no changes in peripheral blood lymphocytes were observed. Given that responses were observed in just over half of study participants, it will be important in future studies to identify the factors determining individual responsiveness or resistance to intermittent fasting interventions.

Relatively few clinical trials are investigating the effects of intermittent fasting alone or in combination with chemo-immunotherapy in cancer patients with active disease (Table 2). One trial (NCT03700437) is investigating the ability of a fasting-mimicking diet to improve combined chemotherapy/ICB (carboplatin/pemetrexed and pembrolizumab [anti-PD-1]) outcomes in patients with advanced non-small cell lung cancer. Subjects are being provided Chemolieve^®^ (L-Nutra, Los Angeles, CA), a plant-based, ~300 kcal per day dietary intervention ~72 h prior to and ~24 h post chemo-immunotherapy for the first four cycles of treatment. Another trial (NCT03162289) is investigating the potential interaction between chemotherapy and intermittent energy restriction (fasting with the exception of ~300–400 kcal/day of vegetable juices immediately before and after each chemotherapy cycle) in breast and ovarian cancer patients. Future clinical trials will need to determine not only the best type of intermittent fasting approach to combine with specific cancer therapies, but also the relative timing of intermittent fasting or fasting-mimicking interventions vs. administration of ICB or other immunotherapies.

## Ketogenic DIET

There also is interest in using other dietary interventions, such as the ultra-low carbohydrate ketogenic diet (Table 1), to slow cancer progression and/or improve treatment efficacy. Ketogenic diets do not directly restrict calories or require periods of fasting but restrict carbohydrate to typically <5% of energy intake. Given their ability to heavily shift metabolism toward fat oxidation and drive the generation of ketone bodies, which also suppress appetite, they effectively reduce plasma glucose concentrations in cancer-free individuals (37). Thus, there is a growing interest in administering ketogenic diets to cancer patients, with the goal of leveraging reduced systemic glucose as a means of inhibiting tumor cell proliferation (38–40), possibly through the induction of oxidative cellular damage and/or increases in cytokine production and cytolysis via tumor-reactive CD8^+^ T cells (41, 42).

### Pre-clinical Findings

Numerous pre-clinical studies show beneficial anti-cancer effects of the ketogenic diet independent of therapy or in combination with radiotherapy or chemotherapy (41–45); however, to our knowledge, no preclinical study has reported the effects of ketogenic diet on immunotherapy outcomes. Thus, it is not clear at this time whether the degree of glucose limitation that occurs during the use of a ketogenic diet will inhibit or promote anti-tumor immunity and immunotherapeutic outcomes.

### Clinical Findings

The beneficial effects of the ketogenic diet observed in pre-clinical models have not translated clinically to all tumor types; however, there are promising results demonstrating the potential of the ketogenic diet to be used as an adjuvant for targeted cancer therapies, as recently reviewed by Klement (46). Ketogenic diet administration in women with ovarian or endometrial cancer, some of whom were treated with chemotherapy, showed beneficial effects on overall physical function and serum insulin, but this study was under-powered to evaluate effects on treatment outcomes or overall survival (40). However, some studies report a decrease in insulin sensitivity and increase in the inflammatory marker high-sensitivity C-reactive protein (hs-CRP) (47), raising some concerns. To our knowledge, the ketogenic diet has not been combined with immunotherapy in any clinical trials (Table 2). Given the growing lay interest in this diet as a weight loss and diabetes intervention, clinical trials designed to determine its impact on cancer treatment outcomes are urgently needed.

## Calorie Restriction Mimetics and Anti-Hyperglycemic Agents

Because prolonged CR is difficult to maintain and negatively impacts lean mass, there also is mounting interest in CR “mimetics,” or compounds that produce many of the same benefits as CR without the need to reduce caloric intake (Table 1). Many CR mimetics are anti-hyperglycemic agents themselves, such as the diabetes medications metformin and acarbose, and the natural phenolic acid compound resveratrol (48–51). As summarized below, multiple studies have begun to examine whether repurposing these anti-hyperglycemic agents to target the metabolic pathways used by cancer cells and/or to reverse metabolic defects within anti-tumor immune populations can improve immunotherapy treatment outcomes.

### Pre-clinical Findings

In murine models, the CR mimetic hydroxycitrate, a citric acid derivative and inhibitor of ATP citrate lyase, has shown similar efficacy to short-term fasting in mice with tumors. One study demonstrated that hydroxycitrate enhanced the ability of the anthracycline chemotherapeutic mitoxantrone to retard subcutaneous tumor outgrowth in models of fibrosarcoma and breast cancer (31). In that study, improvements in outcomes were dependent upon CD8^+^ T cells and decreases in regulatory T cell infiltration into tumors, as well as heightened tumor cell autophagy.

Metformin is a commonly-prescribed anti-hyperglycemic agent that has multiple mechanisms of action that include the activation of AMP-activated protein kinase (AMPK), the inhibition of Complex I of the mitochondrial respiratory chain, and the reduction in hepatic gluconeogenesis (52). Several pre-clinical studies have combined metformin (53, 54) or other anti-diabetic drugs such as phenoformin (55) with ICB. These combination therapies reduced myeloid-derived suppressor cell accumulation in tumors while simultaneously increasing proliferation and cytokine secretion in intra-tumoral CD8^+^ T cells, leading to a net reduction in tumor outgrowth. An explanation for these improvements in T cell function is the finding that metformin treatment of mice with B16 melanoma tumors inhibited tumor cell metabolism (both oxidative phosphorylation and glycolysis) but concurrently enhanced endogenous CD8^+^ T cell metabolism (oxidative phosphorylation) and cytokine production (53). To our knowledge, only one pre-clinical study has examined the effects of metformin on CD19-CAR T cells. In this study, metformin was found to inhibit CD19-CAR T cell proliferation and cytotoxicity, and induce apoptosis of these cells *in vitro*; metformin also suppressed the cytotoxicity of CD19-CAR T cells *in vivo* (56). It remains to be determined if the metabolic programming of genetically engineered, *ex vivo* expanded T cells differs from that present during endogenous T cell responses. Nevertheless, this is an area of intense research (57, 58) that warrants further investigation, as these results suggest that metformin may impede CD8^+^ T cell effector functions in some therapeutic settings.

Another popular CR mimetic is resveratrol, a food-derived compound capable of suppressing multiple signaling pathways related to cell proliferation, genome instability, and tumor angiogenesis while enhancing immunosurveillance mechanisms (59, 60). Data from *in vitro* (61) and *in vivo* (49, 62) studies demonstrate that resveratrol induces apoptosis in cancerous cells by suppressing the anti-apoptotic B cell lymphoma 2 (Bcl-2) family of regulator proteins and inhibiting nuclear factor kappa-light-chain-enhancer of activated B cells (NF-KB) and activator protein-1 (AP-1). An immunomodulator (59) and chemosensitizing agent (63, 64), resveratrol also improves interleukin (IL)-2-based immunotherapy outcomes in models of melanoma (65) and neuroblastoma (66). These improvements are mediated, in part, by increased immune cell infiltration into the tumor microenvironment, blunted expansion of regulatory T cells, and enhanced *in vitro* susceptibility of tumor cells to the cytotoxicity of IL-2-activated killer cells. Overall, these pre-clinical studies suggest that resveratrol both improves anti-tumor immunity and directly increases tumor susceptibility to immune-mediated killing.

### Clinical Findings

In human studies, epidemiological analyses suggest that chronic administration of some types of anti-hyperglycemic medications (e.g., biguanides such as metformin) or natural polyphenols (such as resveratrol) can reduce cancer risk (50, 67, 68), but few studies have investigated their ability to be combined with immunotherapy. One retrospective cohort study (*n* = 55 patients) observed trending improvements in overall and progression-free survival in study participants with metastatic malignant melanoma who receive the metformin in combination with ICB (ipilimumab [anti-cytotoxic T-lymphocyte-associated protein 4 or CTLA-4], nivolumab [anti-PD-1], and/or pembrolizumab); however, these changes failed to reach statistical significance compared to ICB alone (69). Another trial is currently investigating metformin in combination with immunotherapy in non-small cell lung cancer (NCT03048500). No clinical studies have reported on the ability of hydroxycitrate or resveratrol to impact ICB efficacy; however, trials in cancer-free (70–72) and tumor-bearing (73–75) subjects suggest that resveratrol can alter systemic metabolites to improve T cell function and favor an anti-cancer response. Clearly, there is a need for larger retrospective analyses and multi-center prospective studies to evaluate the potential benefits of combining anti-hyperglycemic agents or CR mimetics, such as hydroxycitrate, metformin, or resveratrol, with ICB or other types of immunotherapy.

## Limitations

As clinical data on glucose-modulating interventions discussed above are sparse or non-existent, there is a pressing need for safety and efficacy data from both animal studies and carefully conducted, prospective clinical trials in cancer patients receiving glucose-modulating interventions in the context of ICB or other immunotherapies. In particular, before proceeding to clinical trials, it is important to better understand the mechanisms of interaction of glucose-modulating interventions with the immune system. Because this information is currently lacking, we caution that there is insufficient data to justify combining glucose-modulating lifestyle-based interventions and/or anti-hyperglycemic agents with immunotherapy in advanced cancer patients. However, this is a promising area of research that warrants further investigation, particularly as several of the lifestyle interventions discussed here, such as intermittent fasting or use of over-the-counter calorie restriction mimetics, are gaining popularity in the lay public and may therefore be adopted by some cancer patients in an attempt to improve their health and treatment outcomes.

## Conclusion and Outlook

Here, we have summarized existing pre-clinical murine and human subject data regarding the effects of lowering glucose availability in tumors, either via lifestyle modifications or the use of anti-hyperglycemic agents, on anti-tumor immunity and immunotherapy outcomes. Although the data are preliminary and should be interpreted cautiously, most studies indicate that such glycemic-targeting interventions do not appear to negatively compromise anti-tumor immunity in the context of ICB, and several studies provide evidence of *improved* T cell function and number, with simultaneous reductions in tumor cell proliferation. It is important to note that these beneficial trends may be reversed in the context of CAR T cell therapies. Future pre-clinical studies should seek to identify the mechanisms by which glycemic pathways both directly and indirectly modulate the metabolism, function, and viability of cancer cells vs. CD8^+^ effector T cells and other leukocyte subsets. Moreover, since many of these lifestyle and pharmacologic interventions are pleiotropic, it will also be important to determine whether there are additional immunomodulating or anti-proliferative effects induced through glucose-independent mechanisms.

## Author Contributions

All authors listed have made a substantial, direct and intellectual contribution to the work, and approved it for publication.

### Conflict of Interest Statement

The authors declare that the research was conducted in the absence of any commercial or financial relationships that could be construed as a potential conflict of interest.
